# Temporal and Spatial Clustering of Intracerebral Hemorrhage in Cerebral Amyloid Angiopathy

**DOI:** 10.1212/WNL.0000000000209770

**Published:** 2024-08-16

**Authors:** Simon Fandler-Höfler, Gareth Ambler, Gargi Banerjee, Philip S. Nash, Lena Obergottsberger, Gerit Wünsch, Christian Kiss, Linda Fabisch, Markus Kneihsl, Wenpeng Zhang, Hatice Ozkan, Martina Locatelli, Yang Du, Larysa Panteleienko, Rom Mendel, Kitti Thiankhaw, Robert J. Simister, Hans Rolf Jäger, Christian Enzinger, Thomas Gattringer, David J. Werring

**Affiliations:** From the Department of Neurology (S.F.-H., L.O., C.K., L.F., M.K., C.E., T.G.), Medical University of Graz, Austria; Stroke Research Centre (S.F.-H., G.B., P.S.N., W.Z., H.O., M.L., Y.D., L.P., R.M., K.T., R.J.S., D.J.W.), Department of Brain Repair & Rehabilitation, UCL Queen Square Institute of Neurology; Department of Statistical Science (G.A.), University College London; MRC Prion Unit at UCL (G.B.), Institute of Prion Diseases, London, United Kingdom; Institute for Medical Informatics, Statistics and Documentation (G.W.), and Division of Neuroradiology, Vascular and Interventional Radiology (M.K.), Department of Radiology, Medical University of Graz, Austria; and Neuroradiological Academic Unit (H.R.J.), Department of Brain Repair & Rehabilitation, UCL Queen Square Institute of Neurology, London, United Kingdom.

## Abstract

**Objectives:**

Cerebral amyloid angiopathy (CAA)–associated lobar intracerebral hemorrhage (ICH) has a high risk of recurrence, but the underlying mechanisms remain uncertain. We, therefore, aimed to characterize patterns of recurrent ICH.

**Methods:**

We investigated early recurrent ICH (≥1 recurrent ICH event within 90 days of the index event) and ICH clusters (≥2 ICH events within 90 days at any time point) in 2 large cohorts of consecutive patients with first-ever ICH and available MRI.

**Results:**

In 682 included patients (median age 68 years, 40.3% female, median follow-up time 4.1 years), 18 (2.6%) had an early recurrent ICH, which was associated with higher age and CAA. In patients with probable CAA, the risk of early recurrent ICH was increased 5-fold within the first 3 months compared with during months 4–12 (hazard ratio 5.41, 95% CI 2.18–13.4) while no significant difference was observed in patients without CAA. In patients with an ICH cluster, we observed spatial clustering (recurrent ICH within close proximity of index ICH in 63.0%) and a tendency for multiple sequential hemorrhages (≥3 ICH foci within 3 months in 44.4%).

**Discussion:**

Our data provide evidence of both temporal and spatial clustering of ICH in CAA, suggesting a transient and localized active bleeding-prone process.

## Introduction

Intracerebral hemorrhage (ICH) is a severe form of stroke, leading to high mortality and disability.^1^ Survivors of ICH have a substantially elevated risk of recurrent ICH, which has an estimated mortality rate of 50%.^2^ An important factor associated with a high risk of recurrent ICH is underlying cerebral amyloid angiopathy (CAA),^3^ which has a 7.4%–8.5% annual recurrence risk.^3,4^ Patients with CAA and cortical superficial siderosis (CSS) are at particularly high risk of ICH^5,6^ while a high number of cerebral microbleeds (CMBs) is related to increased risk in both patients with and without CAA.^3^

Although CAA is usually considered to be a chronic, progressive small vessel disease (SVD), in clinical practice, patients with CAA-associated ICH frequently have early recurrences; this observation is supported by data from cohort studies demonstrating a high recurrence risk within the first months,^4,7-11^ but we are not aware of systematic investigations quantifying this phenomenon. Characterizing temporal (and spatial) patterns of early ICH recurrence in CAA could inform mechanistic hypotheses and identify features associated with periods of elevated disease activity and ICH risk with potential relevance for developing rational prevention strategies.

## Methods

We used data from 2 large cohort studies consisting of consecutive patients with spontaneous ICH: the Stroke Investigation Group in North and Central London registry with prospective assessment of patients between 2015 and 2021 and the Graz ICH cohort with standardized retrospective assessment of clinical data from 2008 to 2021. Detailed descriptions of both cohorts have been reported previously.^4,12,13^ For both cohorts, MRI was performed as a standard of care in the inpatient workup of ICH etiology during the entire study period, unless there were contraindications or very poor prognosis due to severe ICH.

We screened all patients with first-ever imaging-confirmed ICH from these 2 cohorts with available baseline MRI within 30 days of index ICH. We excluded patients with macrovascular, structural, or other secondary causes of ICH or who were lost to follow-up due to living outside the catchment area of our hospital system. A study flowchart is shown in eFigure 1.

MRI protocols included T2-weighted fluid-attenuated inversion recovery, T2-weighted images, diffusion-weighted images, and susceptibility-weighted imaging or T2* gradient-echo images. MRI rating was performed using our previously reported standardized approach,^4^ which includes assessment of hematoma location and extension and markers of SVD and classification of etiology, described in detail in the supplement.

Follow-up regarding recurrent ICH was performed using all available electronic health records for both centers and surrounding hospital care systems. All potential recurrent ICH events were evaluated through detailed case-by-case review including neuroimaging confirmation.

The main outcomes were early recurrent ICH (≥1 recurrent ICH event within 90 days of the index ICH) and ICH clusters (defined as ≥2 ICH events within 90 days occurring at any time point during the follow-up period). We further investigated spatial clustering of ICH, which we defined as the epicenter of a recurrent ICH within 3 cm of the border of the index hematoma, irrespective of which lobe was affected.

We performed statistical analysis using STATA (version 18; StataCorp LLC, College Station, TX). In addition to common methods of descriptive and inferential statistics (eMethods), we used piecewise exponential survival models to compare differences in risk between different time windows, visualizing results using flexible parametric survival (Royston-Parmar) models.^14^

### Standard Protocol Approvals, Registrations, and Patient Consents

The study was approved by the ethics committee of the Medical University of Graz and by the University College Hospital NHS Foundation Trust Governance Review Board as a continuous service evaluation of a comprehensive clinical care program. As a retrospective cohort study using deidentified routinely collected clinical data, the need for individual informed consent was waived.

## Results

We included 682 patients with a median age of 68 years (interquartile range [IQR] 57–77), and 40.3% were female. The median follow-up duration was 4.1 years (IQR 1.8–7.7). 79 patients (11.6%) had a recurrent ICH during the observation period, 18 (2.6%) had an early recurrent ICH within 90 days of the index ICH (Table), and 9 additional patients had an ICH cluster later in the observation period.

**Table:** Clinical Characteristics of Study Patients Categorized by ICH Recurrence

	Early recurrent ICH (within 90 d) (n = 18, 2.6%)	No early recurrent ICH (n = 664)		*p* Values (early vs no early recurrent ICH)
		Other (later) recurrent ICH (n = 61, 8.9%)	No ICH recurrence (n = 603, 88.4%)	
Clinical data				
Age, y, median (IQR)	78 (65–82)	70 (64–78)	67 (57–76)	<0.001
Male sex, n (%)	9 (50)	37 (60.7)	360 (59.7)	0.40
Arterial hypertension, n (%)	14 (77.8)	45 (73.8)	459 (76.1)	0.85
Diabetes mellitus, n (%)	0 (0)	12 (19.7)	100 (16.6)	0.06
Systolic blood pressure (admission), mean ± SD	163 ± 31	171 ± 37	176 ± 34	0.21
Diastolic blood pressure (admission), mean ± SD	87 ± 16	94 ± 16	97 ± 19	0.12
Hematoma location, n (%)				<0.001
Lobar	16 (88.9)	43 (70.5)	241 (40.0)	
Deep	2 (11.2)	16 (26.2)	316 (52.4)	
Cerebellar	0	2 (3.3)	46 (7.6)	
MRI findings				
Index ICH volume, median (IQR)	15.8 (6.3–31.3)	9.4 (3.6–23.2)	7.6 (2.8–20.1)	0.04
Subarachnoid extension of ICH, n (%)	12 (66.7)	27 (44.3)	106 (17.6)	<0.001
Concomitant intraventricular hemorrhage, n (%)	4 (22.2)	14 (23.0)	156 (25.9)	0.74
Cortical superficial siderosis, any, n (%)	13 (72.2)	29 (47.5)	57 (9.5)	<0.001
Disseminated cortical superficial siderosis, n (%)	10 (55.6)	17 (27.9)	29 (4.8)	<0.001
Microbleeds, any, n (%)	16 (88.9)	51 (83.6)	375 (62.2)	0.03
Microbleed number, median (IQR)	6 (2–24)	5 (1–26)	1 (0–7)	<0.001
Lobar microbleed number, median (IQR)	5 (2–23)	3 (1–21)	0 (0–3)	<0.001
Deep microbleed number, median (IQR)	0 (0–0)	0 (0–2)	0 (0–2)	0.24
Moderate-to-severe white matter hyperintensities (Fazekas 2–3), n (%)	12 (66.7)	39 (63.6)	299 (49.6)	0.19
Lacunes, any, n (%)	2 (11.1)	14 (23.0)	187 (31.0)	0.08
>20 enlarged perivascular spaces (centrum semiovale), n (%)	13 (76.5)	28 (50.0)	225 (40.7)	0.004
>20 enlarged perivascular spaces (basal ganglia), n (%)	4 (23.5)	8 (14.3)	147 (26.1)	0.89
Diffusion-weighted imaging positive lesion, n (%)	4 (22.2)	7 (11.7)	86 (14.5)	0.34
ICH etiology, n (%)				<0.001
Cerebral amyloid angiopathy	14 (77.8)	32 (52.5)	101 (16.7)	
Arteriolosclerosis	1 (5.6)	5 (8.2)	157 (26.0)	
Mixed small vessel disease	3 (16.7)	24 (39.3)	244 (40.5)	
Cryptogenic	0	0	101 (16.7)	

Abbreviations: ICH = intracerebral hemorrhage; IQR = interquartile range.

Patients with an early recurrent ICH were older; more frequently had probable CAA; and had higher rates of lobar hemorrhage, subarachnoid extension of ICH, CSS, lobar CMB, and severely enlarged perivascular spaces in the centrum semiovale (Table).

### Patterns in Temporal Risk of ICH Recurrence

**Figure 1:**
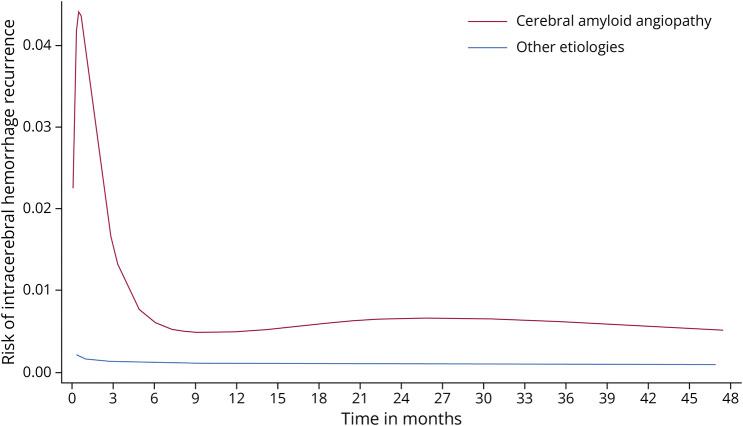
Temporal Changes in Risk of Recurrent Intracerebral Hemorrhage

The ICH recurrence rate in the study population was elevated in the first 3 months after first-ever ICH, compared with during months 4–12 (hazard ratio 4.68, 95% CI 2.21–9.90, *p* < 0.001, eTable 1), a phenomenon almost entirely accounted for by patients with CAA (hazard ratio 5.41, 95% CI 2.18–13.4). For other ICH etiologies, there was no statistically significant difference in temporal patterns of recurrence risk (*p* < 0.10). Figure 1 depicts temporal changes in ICH recurrence.

### Patterns of Patients With ICH Clusters

In addition to the 18 patients with early recurrent ICH, 9 patients had an ICH cluster later in the observation period. When considering all 27 ICH clusters in total, 23 (85.2%) had probable CAA.

In patients with an ICH cluster, the median time to recurrent ICH was 48 days (IQR 19–76 days). Patients with early recurrent ICH frequently had multifocal simultaneous hemorrhages at index presentation or recurrence (9/27, 33.3%) and 3 or more hemorrhages within 90 days (12/27, 44.4%). 17 of 27 patients (63.0%) with an ICH cluster had a recurrent ICH within close proximity of the index ICH. 15 of 20 patients (75%) with available MRI at the time of index ICH demonstrated acute cSAH or cortical superficial siderosis at what would become the site of their recurrent ICH event.

**Figure 2:**
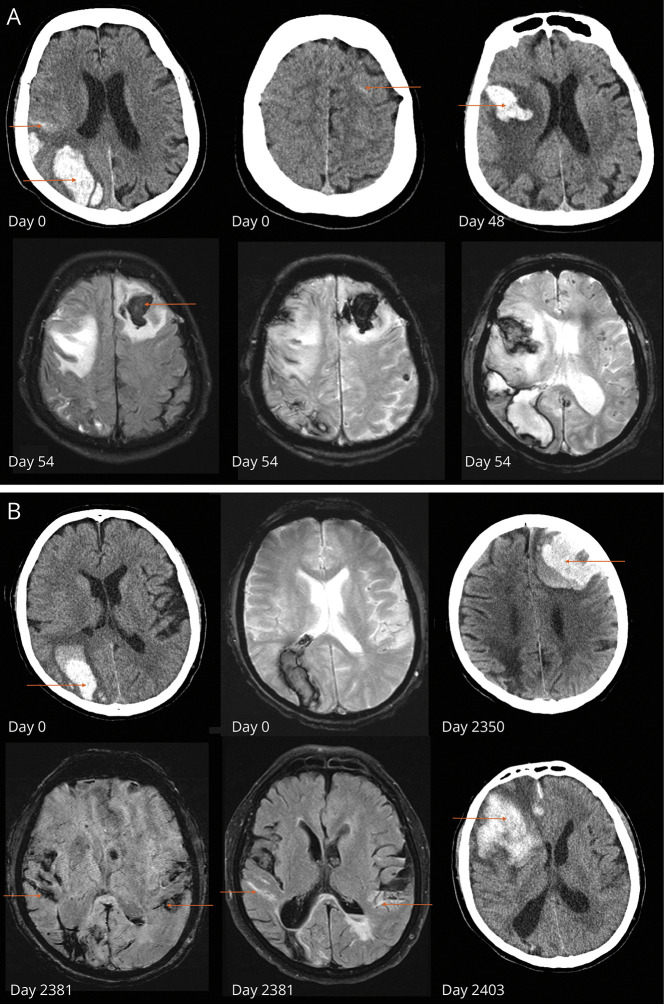
Neuroimaging Findings in 2 Exemplary Patients With ICH Clusters (A) A patient in their seventies with index right parietal intracerebral hemorrhage. On index CT, 2 additional separate foci of convexity subarachnoid hemorrhage were visible (right parietal, left frontal). 48 days after the index ICH, the patient suffered a recurrent ICH in the right frontal lobe. On MRI after clinical worsening 6 days later, an additional large left frontal ICH was visible, as were disseminated cortical superficial siderosis and numerous lobar microbleeds. This patient did not have any further recurrent ICH over a follow-up period of 9 years (with several CT scans performed in the follow-up period). (B) Another patient in their seventies with right occipital intracerebral hemorrhage. T2* MRI sequences did not show additional chronic hemorrhagic lesions. 6 years later, the patient suffered a left frontal hemorrhage. After initial stabilization, MRI depicted bilateral parietal acute convexity subarachnoid hemorrhage and extensive cortical superficial siderosis. 53 days after the second ICH, the patient had a large right frontal ICH and died shortly thereafter. ICH = intracerebral hemorrhage.

13 of 27 patients (48.1%) died within 3 months of ICH clusters. Of the remaining 14 surviving patients (median follow-up 4 years after the last event of ICH cluster; total follow-up 54 patient-years), there were only 2 recurrent ICH events (one after 9 months, the other after 6 years in separate patients). Most ICH clusters were followed by quiescent periods. Two examples of patient courses and related neuroimaging are shown in Figure 2, and individual time lines for all patients with CAA-related ICH clusters are displayed in eFigure 2.

## Discussion

In this observational study investigating temporal and spatial patterns of ICH recurrence in 2 large cohorts of consecutive patients with first-ever ICH, we observed a markedly increased risk of ICH recurrence in the first 3 months after the index event; this phenomenon was almost entirely specific to patients with CAA. This observation may have clinical and mechanistic implications: first, it implies an “active” bleeding-prone phase of disease, which may provide a promising window for intervention to reduce recurrent hemorrhage risk; second, the high rate of recurrent ICH in close anatomical proximity to the index ICH suggests a locally active intracranial vascular process. While the underlying mechanisms remain uncertain, it has recently been hypothesized that focal inflammatory processes may have a role in CAA-related bleeding from leptomeningeal arteries.^15^

The strengths of this study include the combination of 2 independent and large “real-world” cohorts of consecutive patients with ICH, the detailed assessment of underlying SVD using MRI, and systematic assessment of recurrent events. However, variability between used MRI scanners and sequences might have led to differences in the accuracy of SVD assessment. The total number of patients with ICH clusters was limited, and the recurrence rate might have been underestimated by the restriction of the study cohort to patients with available MRI (excluding some patients with severe ICH or very early ICH recurrence) and usage of electronic records to determine ICH recurrence, which although being extensive, might have missed some individual events.

## Appendix Authors

**Table:**

Name	Location	Contribution
Simon Fandler-Höfler, MD, PhD	Department of Neurology, Medical University of Graz, Austria; Stroke Research Centre, Department of Brain Repair & Rehabilitation, UCL Queen Square Institute of Neurology, London, United Kingdom	Drafting/revision of the manuscript for content, including medical writing for content; major role in the acquisition of data; study concept or design; analysis or interpretation of data
Gareth Ambler, PhD	Department of Statistical Science, University College London, United Kingdom	Drafting/revision of the manuscript for content, including medical writing for content; analysis or interpretation of data
Gargi Banerjee, BMBCh, PhD	Stroke Research Centre, Department of Brain Repair & Rehabilitation, UCL Queen Square Institute of Neurology; MRC Prion Unit at UCL, Institute of Prion Diseases, London, United Kingdom	Drafting/revision of the manuscript for content, including medical writing for content
Philip S. Nash, MBBS, MSci	Stroke Research Centre, Department of Brain Repair & Rehabilitation, UCL Queen Square Institute of Neurology, London, United Kingdom	Drafting/revision of the manuscript for content, including medical writing for content; major role in the acquisition of data
Lena Obergottsberger, MD	Department of Neurology, Medical University of Graz, Austria	Major role in the acquisition of data
Gerit Wünsch, PhD	Institute for Medical Informatics, Statistics and Documentation, Medical University of Graz, Austria	Major role in the acquisition of data
Christian Kiss, MD	Department of Neurology, Medical University of Graz, Austria	Drafting/revision of the manuscript for content, including medical writing for content
Linda Fabisch, MD	Department of Neurology, Medical University of Graz, Austria	Major role in the acquisition of data
Markus Kneihsl, MD, PhD	Department of Neurology, and Division of Neuroradiology, Vascular and Interventional Radiology, Department of Radiology, Medical University of Graz, Austria	Drafting/revision of the manuscript for content, including medical writing for content
Wenpeng Zhang, MSc	Stroke Research Centre, Department of Brain Repair & Rehabilitation, UCL Queen Square Institute of Neurology, London, United Kingdom	Major role in the acquisition of data
Hatice Ozkan, PhD	Stroke Research Centre, Department of Brain Repair & Rehabilitation, UCL Queen Square Institute of Neurology, London, United Kingdom	Major role in the acquisition of data
Martina Locatelli, MD	Stroke Research Centre, Department of Brain Repair & Rehabilitation, UCL Queen Square Institute of Neurology, London, United Kingdom	Major role in the acquisition of data
Yang Du, MD	Stroke Research Centre, Department of Brain Repair & Rehabilitation, UCL Queen Square Institute of Neurology, London, United Kingdom	Major role in the acquisition of data
Larysa Panteleienko, MD	Stroke Research Centre, Department of Brain Repair & Rehabilitation, UCL Queen Square Institute of Neurology, London, United Kingdom	Major role in the acquisition of data
Rom Mendel, MD	Stroke Research Centre, Department of Brain Repair & Rehabilitation, UCL Queen Square Institute of Neurology, London, United Kingdom	Major role in the acquisition of data
Kitti Thiankhaw, MD	Stroke Research Centre, Department of Brain Repair & Rehabilitation, UCL Queen Square Institute of Neurology, London, United Kingdom	Major role in the acquisition of data
Robert J. Simister, PhD	Stroke Research Centre, Department of Brain Repair & Rehabilitation, UCL Queen Square Institute of Neurology, London, United Kingdom	Study concept or design
Hans Rolf Jäger, MD	Neuroradiological Academic Unit, Department of Brain Repair & Rehabilitation, UCL Queen Square Institute of Neurology, London, United Kingdom	Drafting/revision of the manuscript for content, including medical writing for content; study concept or design
Christian Enzinger, MD, PhD	Department of Neurology, Medical University of Graz, Austria	Study concept or design
Thomas Gattringer, MD, PhD	Department of Neurology, Medical University of Graz, Austria	Drafting/revision of the manuscript for content, including medical writing for content; study concept or design
David J. Werring, MBBS, PhD	Stroke Research Centre, Department of Brain Repair & Rehabilitation, UCL Queen Square Institute of Neurology, London, United Kingdom	Drafting/revision of the manuscript for content, including medical writing for content; study concept or design; analysis or interpretation of data
